# Functional Change in the Caudal Pontine Reticular Nucleus Induced by Age-Related Hearing Loss

**DOI:** 10.1155/2018/8169847

**Published:** 2018-04-26

**Authors:** Ning Zhao, Ana'am Alkharabsheh, Fei Xu, Wei Sun

**Affiliations:** ^1^Department of Otolaryngology–Head and Neck Surgery, The First Affiliated Hospital of China Medical University, Shenyang 110001, China; ^2^Center for Hearing & Deafness, Department of Communicative Disorders and Science, State University of New York at Buffalo, 3435 Main Street, Buffalo, NY 14214, USA; ^3^Department of Hearing and Speech Sciences, Zhejiang Chinese Medical University, Hangzhou 310053, China

## Abstract

Increased acoustic startle responses (ASR), which represent reduced uncomfortable loudness level in humans, have been reported in middle-aged C57BL/6J mice with sensorineural hearing loss. Although neural plasticity changes in the central auditory system after the peripheral lesions were suggested to underlie this phenomenon, the neurological cause of exaggerated ASR is still not clear. In this study, the local field potentials and firing rates of the caudal pontine reticular nucleus (PnC), which plays a major role in the ASR pathway, were recorded in 2-month- and 6-month-old C57BL/6 J mice. Consistent with our previous studies, the amplitude of ASR increased, and the threshold of ASR decreased in the 6-month-old mice after developing 20–40 dB hearing loss. The PnC response induced by high-frequency stimuli (>20 kHz) decreased in the 6-month group, whereas the PnC response induced by low-frequency stimuli (<12 kHz) showed a significant increase in the 6-month group compared to the 2-month group. The enhancement of PnC response is similar to the ASR increase found in the 6-month-old C57 mice. Our results suggest that the high-frequency hearing loss caused an increase in PnC sensitivity in the C57 mice which may enhance ASRs.

## 1. Introduction

Age-related hearing loss, one of the most common disorders in the elderly, is predominately associated with sensory receptor cell degeneration. Approximately one in three people in the United States between the ages of 65 and 74 has hearing loss [1]. Age-related hearing loss not only affects the hearing sensitivity but also impairs the central auditory processing the speech signals [2, 3]. Tinnitus and hyperacusis are also commonly found in elderly population which may be due to the progressive increase of age-related hearing loss [4].

Increased acoustic startle responses (ASR) were recorded in animal models of tinnitus induced by high doses of salicylate [5] and noise exposure [6]. ASR is a largely unconscious defensive response to sudden or threatening stimuli recorded in both animals and humans [7, 8]. Recent studies found that ASR amplitude at a given stimulus level increases with decreasing loudness discomfort level (LDL) in human [9]. Therefore, ASR amplitudes may provide an objective indication of LDL, a common audiological evaluation for tinnitus and hyperacusis patients [10].

Enhanced ASRs were reported in C57 mice after they developed moderate high-frequency hearing loss [11, 12]. The loudness augmentation was suggested to be induced by plasticity changes in the central auditory system, for example, possibly by a deficit in centrifugal inhibitory control over the afferent reflex pathways after central neural reorganization [11]. These data suggest that the C57 mouse model may be useful in studying hyperacusis and tinnitus associated with age-related hearing loss. The “startle circuit,” which provides an inhibitory pathway to modulate the ASR amplitude, consists of the auditory nerve, cochlear root neurons, caudal pontine reticular nucleus (PnC), motor neurons in the spinal cord, and the central auditory system, including the inferior colliculus (IC) and auditory cortex (AC) [13, 14]. Our recent study measured neurophysiological changes in the IC of 2-, 6-, and 12-month-old C57 mice and detected increase in sound-evoked activity of the IC in 6-month-old mice. Our data suggested that the hyperexcitability in the IC may be related with enhanced ASR amplitudes. However, the functional changes in PnC neurons, a critical nucleus that controls the amplitude and threshold of ASR, in the C57 mouse model have not been reported. In this study, we recorded local field potentials and firing rates of the PnC in young and aged C57 mice to reveal the physiological source of the enhanced ASR.

## 2. Materials and Methods

### 2.1. Animals

C57BL/6J mice from Jackson Laboratory (Bar Harbor, ME) were used in this study. Six 2-month-old mice (G-2M group) and six 6-month-old mice (G-6M group) were used for ASR and auditory brainstem-evoked response (ABR) test. Twelve mice (six 2-month-old and six 6-month-old mice) were used for extracellular recordings of PnC neurons.

All protocols were approved by the University at Buffalo Institutional Animal Care and Use Committee (IACUC) and conformed to the guidelines issued by the National Institutes of Health.

### 2.2. ABR Recording

ABRs were recorded in 2-month- and 6-month-old mice in a soundproof room to evaluate their hearing thresholds (Sun et al.). The mice were anesthetized with a mixture of ketamine (100 mg/kg) and xylazine (10 mg/kg), and ABRs were recorded with stainless steel electrodes. TDT System-3 hardware and software (BioSig, Tucker-Davis Technology, FL, USA) were used for ABR recording. For the differential amplifier input, the vertex was used as noninverting (+), the pinna on the stimulating side was used as inverting (−), and the pinna on the contralateral side was used as the ground. Tone bursts at 4, 8, 16, 24, 32, and 48 kHz (5 ms duration, 0.1 ms rise/fall time) were used to elicit the ABR responses. ABR thresholds were obtained for each animal using a step of 5–10 dB SPL to identify the lowest intensity that elicited a response.

### 2.3. ASR Recordings

TDT hardware and custom software were used for recording ASR [5, 15]. Briefly, animals were placed in a small, wire mesh cage mounted on a plexiglass base that rested on a sensitive piezoelectric transducer. The wire mesh (0.5 cm × 0.5 cm) cage (4 cm W × 3.5 cm H × 7-8 cm L) restricted the mouse's movement within a calibrated sound field. The output of the piezo transducer was connected to an A/D converter on an RP2 real-time processor (TDT). The ASR was amplified and filtered (0–1000 Hz) using a low-pass filter (LPF-300, World Precision Instruments, Sarasota, FL, USA). The root mean square (RMS) of ASR was calculated using the custom software. Sound stimuli were presented by a loud speaker (FT28D, Madisound Speaker Components Inc., Middleton, WI, USA) located approximately 28 cm above the mouse's head. The ASR-eliciting stimuli consisted of narrowband noise bursts centered at 8, 12, and 20 kHz (bandwidth 2 kHz, 20 ms) presented at intensities from 60 to 100 dB SPL (10 trials on each condition). The intertrial interval (ISI) was randomly varied from 18 to 23 seconds.

### 2.4. PnC Recordings and Labeling

The PnC neurons were recorded from the mice anesthetized with a mixture of ketamine (100 mg/kg) and xylazine (10 mg/kg, i.p.). The surgical procedure for recording from the PnC was similar to that of the IC recording which has been described in our recent publication [15]. Briefly, the skin over the parietal bone was carefully removed to expose the skull and then treated with 3% hydrogen peroxide (H_2_O_2_). A head-fixing pole attached to the skull was used to firmly hold the mouse's head during the surgery. A 4 × 4 mm region of the cranial bone overlying the dorsocaudal region of the cerebellum was removed to expose the left IC.

A 16-channel microelectrode (NeuroNexus, A1 × 16-5 mm-100e177, Ann Arbor, MI), mounted on a hydraulic manipulator (FHC Inc., Bowdoinham, ME), was inserted into the lateral side of IC towards the PnC. A broadband noise burst (80–90 dB SPL) was used as a search sound to monitor neural responses. First, we found IC responses emerging from the surface to 2.5 mm depth of the brain and then completely disappearing after 3.5 mm. PnC response reemerged at approximately 4-5 mm depth from the surface. Typically, 3–5 penetrations were used to search for PnC responses in each mouse, starting from the lateral side and moving medially while avoiding major blood vessels. The output of the electrode was connected to a 16-channel preamplifier (RA16PA, TDT), and the preamplifier was connected to a digital signal processing module (RZ5, TDT) connected to a computer. A stainless steel electrode inserted into the neck muscle was used as the ground.

Sound stimuli were generated with the TDT System-3 hardware and presented through a multifield magnetic speaker (MF1, TDT). The rate-level function (RLF), excitatory frequency response area (eFRA) of the firing spikes, and the local field potential (LFP) of the PnC neurons were recorded. Noise bursts (0–100 dB SPL) were used to record the RLF. Tone bursts (0–90 dB SPL, 4 to 42 kHz in 20 logarithmically spaced steps) were used to record eFRA at two different interstimulation intervals (ISI) at 1 and 5 seconds. The sound intensity was calibrated using a sound level meter (824, Larson Davis, Depew, NY) with a 1/4-inch condenser microphone (Larson Davis).

During the testing, the mouse's body temperature was maintained at 37°C using a thermally regulated heating pad system (Harvard Apparatus, Cambridge, MA). The multiunit recording was typically completed in 2-3 h, and the pedal withdrawal reflex of the hind limbs was checked every 45 min to assess the anesthetic depth. Supplemental ketamine and xylazine (~0.1 ml) were given as needed to maintain the proper level of anesthesia.

**Figure 1:**
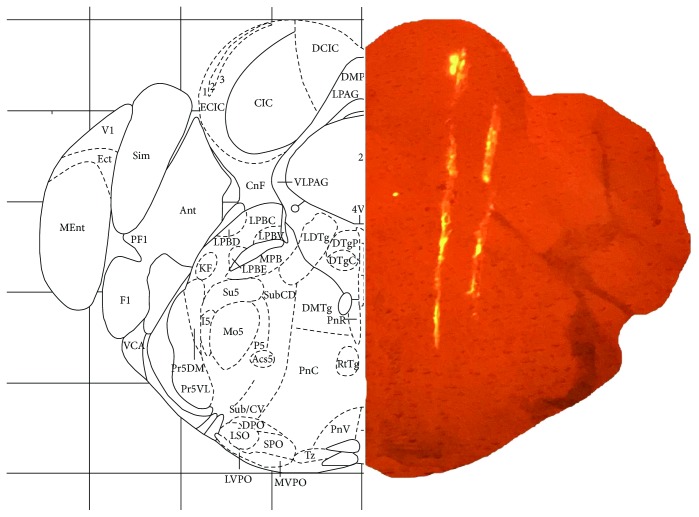
The right image shows the16-channel electrode coated with DiI staining on a mouse brain section (yellow fluorescence). The location of PnC recording was confirmed with the left image which shows the same location from the mouse brain atlas (Paxinos & Watson).

To confirm the recording site of the brain, DiI staining solution (Invitrogen) was applied on the 16-channel electrode prior to the electrophysiological recordings of the PnC. After completion of the recording, the animal was decapitated; the brain was removed from the skull and fixed in 10% formalin for 2 days. Then, the brain was transferred to 30% sucrose solution for 48 h until the brain tissue sunk. Next, the brain was sliced into 40-micron thin sections using a cryostat. DiI-stained electrode insertion was visualized under a fluorescence microscope. Further, DiI-coated electrode tracing of the brain slice (Figure 1) was compared to mouse brain atlas (Paxinos & Watson) to verify the location of the PnC electrophysiological recording.

### 2.5. Statistical Data Analysis

Graph-Pad Prism software (GraphPad Software, San Diego, CA) was used for plotting and statistical analyses unless otherwise noted. Results were presented as mean ± standard error of the mean (SEM). Two-way ANOVAs and Student's *t*-tests were used for the neurophysiological data analysis. Student's *t*-tests were used in the behavioral data analysis. The alpha level was set to *P* < 0.05 for all statistical tests.

## 3. Results

### 3.1. The ABR Assessment of Hearing Loss

**Figure 2:**
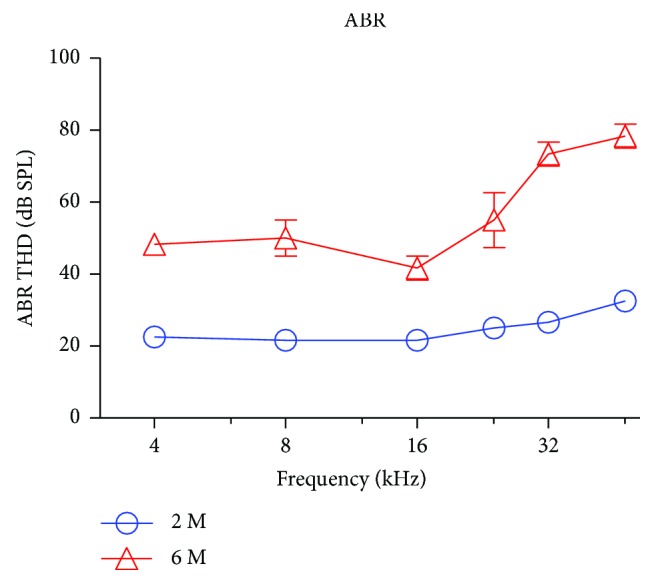
The auditory brainstem response (ABR) thresholds from C57BL/6J mice showed a significant increase with age. The mean ABR thresholds in the 6-month-old group (G-6M, *n* = 6) were significantly higher than those of the 2-month-old group (G-2M, *n* = 6; two-way ANOVA, *P* < 0.0001).

Age-related cochlear hearing loss was evaluated by measuring the ABR thresholds in 2- and 6-month-old mice. Mean ABR thresholds in the 2-month-old C57BL/6J mice (*n* = 6) was 20–25 dB at 4 to 48 kHz. ABR thresholds deteriorated with age (Figure 2). The threshold increases were greater for the high frequencies (>16 kHz) compared to the low frequencies. The mean ABR thresholds of the aged group (G-6M, *n* = 6) were significantly higher than those of the young group (G-2M, *n* = 6) at all of the tested frequencies (two-way ANOVA, *F* = 9.71, *P* < 0.0001). The age-related hearing loss was consistent with previous findings [11, 15].

### 3.2. The ASR Changes with Age

**Figure 3:**
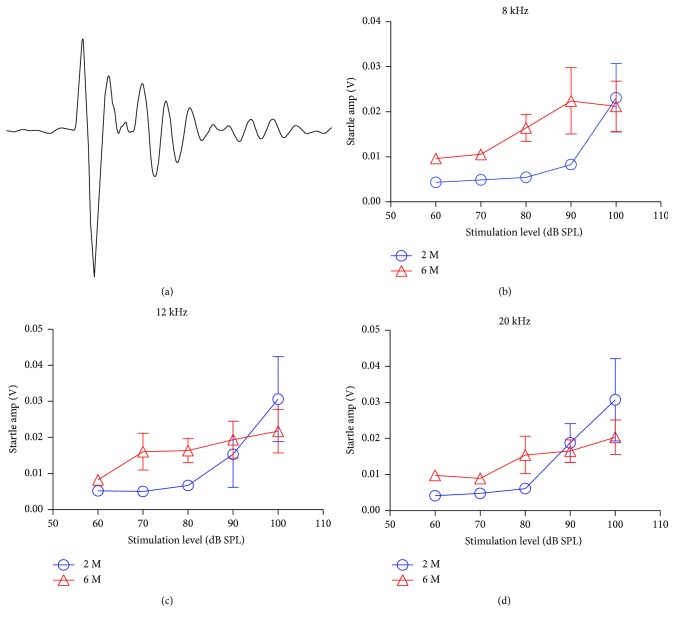
Response of the acoustic startle reflex (ASR) of the 2- and 6-month-old mice elicited by narrowband noise centered at 8, 12, and 20 kHz. (a) A raw startle response recorded from a mouse. (b–d) The startle amplitudes in the 6-month-old mice were significantly larger than those of the 2-month-old mice at 70–80 dB SPL (Student's *t*-test, *P* < 0.05). At high intensities (90–100 dB SPL), the ASR amplitude was similar in the two groups (Student's *t*-test, *P* > 0.05).

To determine the acoustic behavioral consequences of the high-frequency age-related hearing loss, ASR was measured over three consecutive days in different age groups (Figure 3(a)). The RMS values of the ASR amplitude induced by narrowband noise centered at 8, 12, and 20 kHz were measured by a 180 ms window after the onset of sound stimuli (Figures 3(b)–3(d)). The average ASRs as a function of intensity was significantly greater in the 6-month-old mice (*n* = 6) compared to the 2-month group (*n* = 6) at 70 and 80 dB SPL (Student's *t*-test, *P* < 0.05) but no difference at 90 and 100 dB SPL (Student's *t*-test, *P* > 0.05, Figures 3(b)–3(d)).

### 3.3. PnC Response Affected by Age-Related Hearing Loss

**Figure 4:**
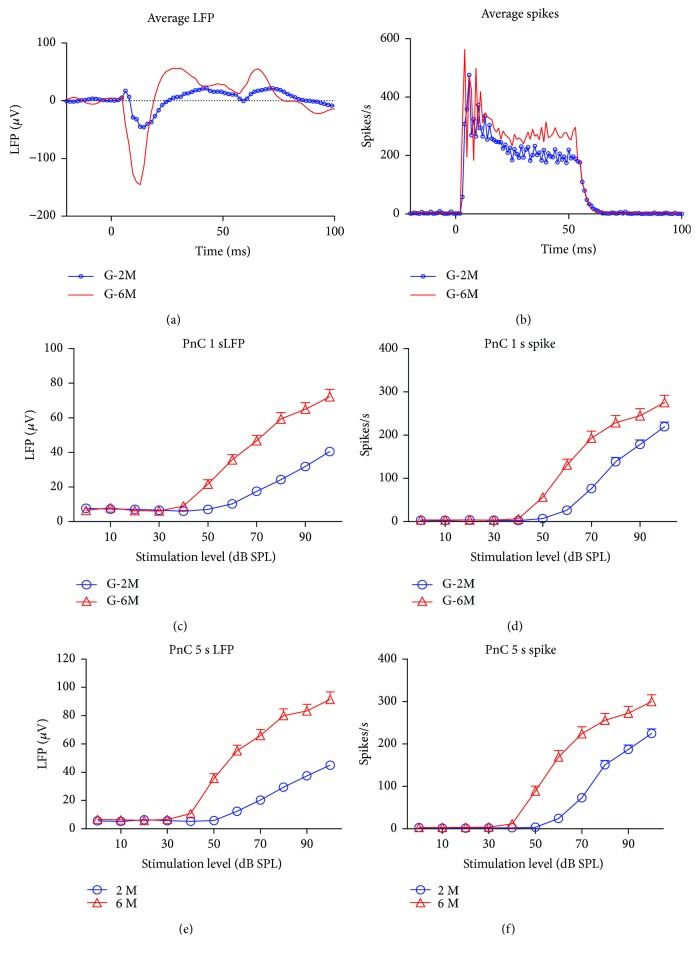
The local field potential (LFP) and spike discharge rates of PnC response elicited by white-noise bursts recorded from the G-2M and G-6M groups. (a) The average LFP at 90 dB SPL recorded from the G-6M group was significantly larger than that from the G-2M group. (b) The average peristimulus time histograms (PSTH, 90 dB SPL) of the 6-month group were also larger than those of the 2 month-old mice. The LFP and firing rate intensity function of the G-6M group were also significantly larger than those of the G-2M group elicited by different interstimulation intervals (ISI) at 1 (c and d) and 5 seconds (e and f) (two-way ANOVA, *P* < 0.001).

To explore the neurophysiological changes in the pathway responsible for ASR, PnC neurons were recorded from the 2-month- and 6-month-old mice. To identify the PnC neurons, a multichannel electrode was advanced into the brain from the lateral side of the IC while noise bursts (50 ms, 70–90 dB SPL) were presented. PnC neurons were typically identified in 1-2 mm from the ventral side of the IC neurons (approximately 4-5 mm below the surface of the brain). The recording site again was confirmed with DiI trace and compared to mouse brain atlas after the recording (Figure 1).  Figure 4 shows the average LFP (Figure 4(a)) and spike discharge rates (Figure 4(b)) of the PnC induced by noise bursts in the G-2M and G-6M group (ISI = 1 second). The RMS value of the PnC responses (50 ms window after sound onset) for each intensity was calculated and compared from the 2-month group to the 6-month group. The average PnC response (*n* = 60) in the 6-month-old mice was significantly higher than that in the 2-month-old mice (*n* = 126) at above 50 dB SPL (Student's *t*-test, *P* < 0.001, Figures 4(c) and 4(d)). The LFP and PSTH of PnC neurons elicited by noise bursts at 5-second ISI are shown in Figures 4(e) and 4(f). Similar to the LFP and PSTH recorded with 1-second ISI, the amplitude of PnC responses in the G-6M group was significantly higher than that in the G-2M group (>50 dB SPL) with 5-second ISI. Comparing the LFP and PSTH recorded with 1 s ISI, the response recorded in 5 s ISI showed no significant changes in the G-2M group. However, the LFP and PSTH recorded in the G-6M group with 5-second ISI were significantly higher than those recorded with short ISI (two-way ANOVA, *P* < 0.001). The amplitude of LFP and PSTH increased by 25% and 10%, respectively.

**Figure 5:**
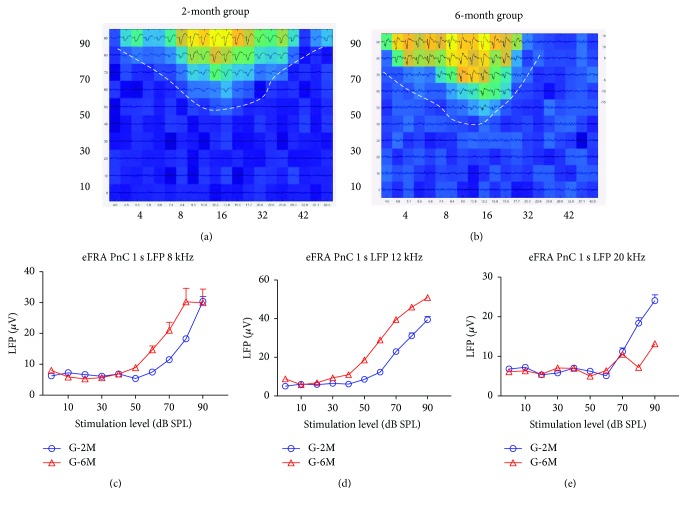
Typical excitatory frequency response area (eFRA) of PnC response in the G-2M and G-6M group (a and b). The threshold of PnC response was about 50–70 dB SPL in the G-2M group. For the 6-month group, there was no response at above 30 kHz. However, at low frequencies (<16 kHz), the threshold of PnC response was slightly lower in the G-6M than in the G-2M group. (c–e) The rate-level function of PnC neurons elicited by tone bursts at 8, 12, and 20 kHz. The amplitude of the PnC response was significantly higher in the G-6M group compared to that in the G-2M group at 8 and 12 kHz at 50–80 dB SPL (Student's *t*-test, *P* < 0.05), whereas the PnC response at 20 kHz in the G-6M group was significantly lower than that in the G-2M group at 80 and 90 dB SPL (Student's *t*-tests, *P* < 0.05).

The typical eFRA in the G-2M and G-6M group is shown in Figures 5(a) and 5(b). The threshold of PnC response was 50 dB SPL or above in the G-2M group. For the G-6M group, there were no responses in the PnC neurons at above 20 kHz. However, at 8 and 12 kHz, the threshold of PnC responses in the G-6M group was about 40 dB SPL, slightly lower than the G-2M group. The RLF of PnC neurons elicited by tone bursts at 8, 12, and 20 kHz is shown in Figures 5(c)–5(e). The amplitudes of the PnC LFPs at 50–80 dB SPL in the G-6M group were significantly higher compared to those in the G-2M group at 8 and 12 kHz (Figures 5(c) and 5(d), two-way ANOVA, *P* < 0.05) but significantly lower at 20 kHz at 80 and 90 dB SPL (Figure 5(e), two-way ANOVA, *P* < 0.05).

## 4. Discussion

The goal of this study was to identify functional changes in the PnC and ASR in age-related hearing loss. There are three major findings of this study: (1) PnC responses recorded in the 6-month-old mice, which showed moderate hearing loss, were significantly higher than those recorded in the 2-month-old mice; (2) the enhancement of PnC response was found predominantly at low frequencies (<12 kHz) which may compensate for the high-frequency hearing loss; and (3) age-related physiological changes of PnC were consistent with the enhancement of ASR and IC function in mice after developing age-related hearing loss [15]. Our results suggest that the sensitivity of PnC neurons at low frequencies increased after developing high-frequency hearing loss, and these physiological changes in the PnC may be directly related to the exaggerated ASR [7].

Typically, the stimulus intensities need to be higher than 70 dB SPL to reliably evoke ASRs [16]. Prepulse inhibition of ASRs is a clinical test for neurological diseases, such as schizophrenia [17], obsessive compulsive disorder [18], and attention deficit/hyperactivity disorder (ADHD) [19]. Recently, ASR amplitude measurement was found to be correlated with reduced LDL in human subjects [9]. ASR may provide an objective indication of LDL since increased ASR amplitudes and decreased thresholds suggest reduced discomfort level to loud sounds. Consistent with previous studies, we found that the 6-month-old C57 mice show enhanced ASR amplitude/reduced threshold after developing hearing loss. These data suggested that decreased loudness discomfort level is correlated with age-related hearing loss which is commonly seen in the elderly [20].

**Figure 6:**
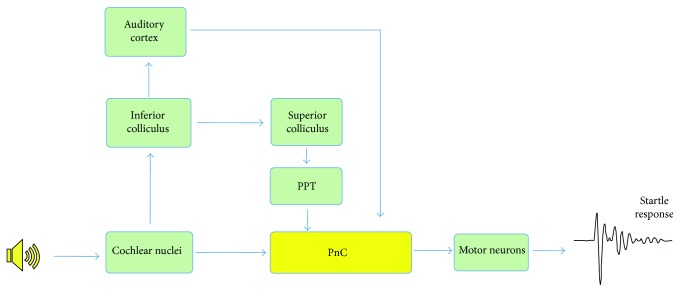
Simplified hypothetical pathway of acoustic startle response and prepulse inhibition (modified from Koch and Schnitzler).

In the startle circuit which is illustrated in Figure 6 (modified from Koch and Schnitzler), PnC neurons are the indispensable sensorimotor interface of the cochleospinal pathway that mediates the ASR. PnC receives direct acoustic input from different nuclei in the central auditory pathway and projects onto the spinal interneurons and motor neurons [13]. Disruption of PnC pathway can dramatically decrease the startle amplitude [21]. Similar to the ASR threshold, PnC neurons also have high firing thresholds and broad frequency tuning indicating that giant PnC neurons have particularly low sensitivity [14]. In this study, we found that PnC thresholds decreased from 70 dB to 50 dB SPL (8 and 12 kHz), and the local field potentials as well as firing rates increased significantly with age. These physiological changes of the PnC neurons were consistent with reduced ASR in age-related hearing loss.

Previous studies reported that the ASR of C57 mice decreased with the onset of hearing loss and increased at low frequencies at 6 months of age [11]. This delayed exaggeration of the ASR with hearing loss may be caused by the deficit in the centrifugal inhibitory control over the afferent reflex pathways after central neural reorganization [11]. PnC neurons receive neural innervations from the auditory system as well as the limbic systems. For example, the IC neurons, particularly the external cortex, have inhibitory projections to the PnC [14]. Our recent study found that the response in the IC increased in the 6-month C57 mice compared to the young mice [15]. This functional change of the IC neurons may interfere with inhibition of ASR [22, 23]. Lingenhohl and Friauf also detected that the activity of amygdala can enhance the response of PnC [14]. Salicylate exposure which causes enhancement of ASR [5] also affects the response of the amygdala and PnC [24, 25].

The central plasticity changes are commonly induced by hearing loss, and the functional changes are also related to the hearing loss frequency. Parham and Willott and Carlson and Willot reported that the ASR amplitude declined for the high-frequency stimuli, and the biggest increase of ASR was at the edge of the high-frequency hearing loss region, that is, 12 kHz [16, 26]. They suggested that this was induced by the strengthening of neural responses to the still audible sounds which may cause the behavioral sound augmentation as well. The argument of ASR may therefore be contributed to the unmasking effect from the hearing loss region [27, 28]. Our current study demonstrated that age-related hearing loss has a direct effect on the function of PnC neurons which may cause exaggerated ASR and loudness changes.

## 5. Conclusions

The response of PnC neurons is enhanced with hearing loss in the 6-month-old C57 mice. These findings support the hypothesis that the responses of PnC neurons would reflect the characteristics of the behavioral ASR. The increased response of PnC could be the neural source of enhanced ASR and reduced LDL associated with age-related hearing loss.

## References

[1] Liu X. Z., Yan D. (2007). Ageing and hearing loss. *The Journal of Pathology*.

[2] Tadros S. F., Frisina S. T., Mapes F., Kim S. H., Frisina D. R., Frisina R. D. (2005). Loss of peripheral right-ear advantage in age-related hearing loss. *Audiology and Neurotology*.

[3] Ohlemiller K. K., Gagnon P. M. (2004). Apical-to-basal gradients in age-related cochlear degeneration and their relationship to “primary” loss of cochlear neurons. *The Journal of Comparative Neurology*.

[4] Longenecker R. J., Chonko K. T., Maricich S. M., Galazyuk A. V. (2014). Age effects on tinnitus and hearing loss in CBA/CaJ mice following sound exposure. *SpringerPlus*.

[5] Sun W., Lu J., Stolzberg D. (2009). Salicylate increases the gain of the central auditory system. *Neuroscience*.

[6] Chen G., Lee C., Sandridge S. A., Butler H. M., Manzoor N. F., Kaltenbach J. A. (2013). Behavioral evidence for possible simultaneous induction of hyperacusis and tinnitus following intense sound exposure. *Journal of the Association for Research in Otolaryngology*.

[7] Li L., du Y., Li N., Wu X., Wu Y. (2009). Top-down modulation of prepulse inhibition of the startle reflex in humans and rats. *Neuroscience & Biobehavioral Reviews*.

[8] Yeomans J. S., Frankland P. W. (1995). The acoustic startle reflex: neurons and connections. *Brain Research Reviews*.

[9] Knudson I. M., Melcher J. R. (2016). Elevated acoustic startle responses in humans: relationship to reduced loudness discomfort level, but not self-report of hyperacusis. *Journal of the Association for Research in Otolaryngology*.

[10] Fournier P., Hebert S. (2013). Gap detection deficits in humans with tinnitus as assessed with the acoustic startle paradigm: does tinnitus fill in the gap?. *Hearing Research*.

[11] Ison J. R., Allen P. D., O’Neill W. E. (2007). Age-related hearing loss in C57BL/6J mice has both frequency-specific and non-frequency-specific components that produce a hyperacusis-like exaggeration of the acoustic startle reflex. *Journal of the Association for Research in Otolaryngology*.

[12] Willott J. F., Kulig J., Satterfield T. (1984). The acoustic startle response in DBA/2 and C57BL/6 mice: relationship to auditory neuronal response properties and hearing impairment. *Hearing Research*.

[13] Koch M., Schnitzler H. U. (1997). The acoustic startle response in rats--circuits mediating evocation, inhibition and potentiation. *Behavioural Brain Research*.

[14] Lingenhohl K., Friauf E. (1994). Giant neurons in the rat reticular formation: a sensorimotor interface in the elementary acoustic startle circuit?. *The Journal of Neuroscience*.

[15] Xiong B., Alkharabsheh A., Manohar S. (2017). Hyperexcitability of inferior colliculus and acoustic startle reflex with age-related hearing loss. *Hearing Research*.

[16] Parham K., Willott J. F. (1988). Acoustic startle response in young and aging C57BL/6J and CBA/J mice. *Behavioral Neuroscience*.

[17] Kohl S., Heekeren K., Klosterkötter J., Kuhn J. (2013). Prepulse inhibition in psychiatric disorders--apart from schizophrenia. *Journal of Psychiatric Research*.

[18] Steinman S. A., Ahmari S. E., Choo T. (2016). Prepulse inhibition deficits only in females with obsessive-compulsive disorder. *Depression and Anxiety*.

[19] Hawk L. W., Yartz A. R., Pelham W. E., Lock T. M. (2003). The effects of methylphenidate on prepulse inhibition during attended and ignored prestimuli among boys with attention-deficit hyperactivity disorder. *Psychopharmacology*.

[20] Chang H. P., Chou P. (2007). Presbycusis among older Chinese people in Taipei, Taiwan: a community-based study. *International Journal of Audiology*.

[21] Davis M., Gendelman D. S., Tischler M. D., Gendelman P. M. (1982). A primary acoustic startle circuit: lesion and stimulation studies. *The Journal of Neuroscience*.

[22] Parham K., Willott J. F. (1990). Effects of inferior colliculus lesions on the acoustic startle response. *Behavioral Neuroscience*.

[23] Leitner D. S., Cohen M. E. (1985). Role of the inferior colliculus in the inhibition of acoustic startle in the rat. *Physiology & Behavior*.

[24] Chen Y. C., Li X., Liu L. (2015). Tinnitus and hyperacusis involve hyperactivity and enhanced connectivity in auditory-limbic-arousal-cerebellar network. *eLife*.

[25] Chen Y. C., Chen G. D., Auerbach B. D., Manohar S., Radziwon K., Salvi R. (2017). Tinnitus and hyperacusis: contributions of paraflocculus, reticular formation and stress. *Hearing Research*.

[26] Carlson S., Willott J. F. (1996). The behavioral salience of tones as indicated by prepulse inhibition of the startle response: relationship to hearing loss and central neural plasticity in C57BL/6J mice. *Hearing Research*.

[27] Wang J., Ding D., Salvi R. J. (2002). Functional reorganization in chinchilla inferior colliculus associated with chronic and acute cochlear damage. *Hearing Research*.

[28] Niu Y., Kumaraguru A., Wang R., Sun W. (2013). Hyperexcitability of inferior colliculus neurons caused by acute noise exposure. *Journal of Neuroscience Research*.

